# MicroRNAs in Long COVID: Key Regulators, Biomarkers, and Therapeutic Targets of Post-SARS-CoV-2 Sequelae

**DOI:** 10.3390/biom16020283

**Published:** 2026-02-11

**Authors:** Rawan Makki, Sondos Kassem-Moussa, Fatima Al Nemer, Rania El Majzoub, Hussein Fayyad-Kazan, Walid Rachidi, Bassam Badran, Mohammad Fayyad-Kazan

**Affiliations:** 1 Laboratory of Cancer Biology and Molecular Immunology, Faculty of Sciences-I, Lebanese University, Beirut 1107 2810, Lebanon; rawan_makki@hotmail.fr (R.M.); sondos.kassem-moussa@ul.edu.lb (S.K.-M.); fatimanemer@hotmail.com (F.A.N.); hfayyadk@gmail.com (H.F.-K.);; 2 Department of Biomedical Sciences, School of Pharmacy, Lebanese International University, Beirut 146404, Lebanon; rania.elmajzoub@liu.edu.lb; 3 University Grenoble Alpes, CEA, Inserm, IRIG, UA13 BGE, Biomics, 38400 Grenoble, France; 4 Department of Natural and Applied Sciences, School of Arts and Sciences, The American University of Iraq-Baghdad, Baghdad, Iraq

**Keywords:** microRNAs, long COVID, pathogenesis, coronavirus, therapy

## Abstract

COVID, or post-acute sequelae of SARS-CoV-2 infection (PASC), is clinically defined by persistent symptoms that endure beyond acute infection and affect multiple organ systems, including the immune, cardiopulmonary, neurological, and metabolic axes. The underlying mechanisms remain poorly resolved, limiting the development of targeted diagnostics and therapeutics. MicroRNAs (miRNAs), as key post-transcriptional regulators of gene expression, control inflammatory networks, antiviral responses, mitochondrial bioenergetics, and fibrotic pathways, all of which are implicated in long COVID pathogenesis. Recent studies show durable changes in circulating miRNA signatures months after recovery from the acute phase, suggesting a role in maintaining chronic immune activation and metabolic dysfunction. Importantly, circulating miRNAs are stable, quantifiable in biofluids, and reflect systems-level dysregulation, positioning them as promising biomarker candidates for patient stratification, symptom clustering, and disease monitoring. Moreover, miRNA-directed interventions, such as mimics and antagomiRs, represent an emerging precision-medicine strategy to correct sustained molecular disturbances. This review summarizes current evidence linking miRNAs to long COVID, highlights their biomarker potential, and discusses therapeutic avenues that may help advance mechanism-based interventions for this globally emerging chronic condition.

## 1. Introduction

Severe acute respiratory syndrome coronavirus 2 (SARS-CoV-2), the causative agent of COVID-19, enters host cells primarily via the angiotensin-converting enzyme 2 (ACE2) receptor, triggering acute respiratory and systemic disease through direct viral cytopathic effects and a robust innate and adaptive immune response. However, while viral replication typically declines and symptoms resolve within weeks for most individuals, an expanding body of evidence demonstrates that viral RNA, subgenomic fragments, and protein antigens can persist long after the acute phase has ended. Sensitive molecular assays have detected SARS-CoV-2 RNA in a range of solid tissues, including liver, kidney, intestine, brain, lung, and skin, months after infection, with subgenomic RNA indicative of recent or ongoing replication in some cases, and persistence in plasma and blood cell compartments, particularly in immunocompromised individuals [1]. Likewise, viral proteins such as spike, S1 subunit, and nucleocapsid have been detected in circulation up to 10–14 months post-infection, suggesting that antigenemia can be prolonged well beyond clinical recovery [2]. Histopathological studies have corroborated these findings, revealing residual viral antigen and RNA within tissues such as the appendix, skin, and breast up to >400 days after symptom onset, often colocalizing with immune cells. These persistent viral elements are hypothesized to contribute to the pathogenesis of long COVID by sustaining chronic immune activation and dysregulation: reservoirs of viral RNA or antigen may continually stimulate innate and adaptive immune responses, leading to prolonged production of pro-inflammatory cytokines, immune cell exhaustion, and tissue-specific inflammation that align with the multisystem symptoms observed in PASC [3]. Furthermore, persistent antigen exposure has been linked with altered T and B cell phenotypes and ongoing cytokine production, creating a milieu of chronic low-grade inflammation that may underlie fatigue, neurocognitive dysfunction, and other long COVID manifestations [4]. Together, these mechanistic insights and empirical data support the concept that viral persistence, whether as genomic fragments, subgenomic RNA, or residual proteins, may play a critical role in initiating and maintaining the inflammatory responses characteristic of long COVID.

Long COVID, or PASC, as defined per WHO criteria, is a complex condition characterized by frequently severe symptoms that manifest weeks, months, or even years after the initial COVID-19 infection has resolved. Current evidence suggests that approximately one-third of individuals who survive acute COVID-19 may develop persistent symptoms consistent with long COVID [5].

Long COVID is associated with all ages and varying degrees of severity [6,7,8]. The highest prevalence is in middle-aged adults falling between 45 and 54 years old [9], with the majority occurring in individuals who did not require hospitalization and experienced a mild acute disease due to the high proportion of mild infections in the total COVID-19 population.

**Figure 1:**
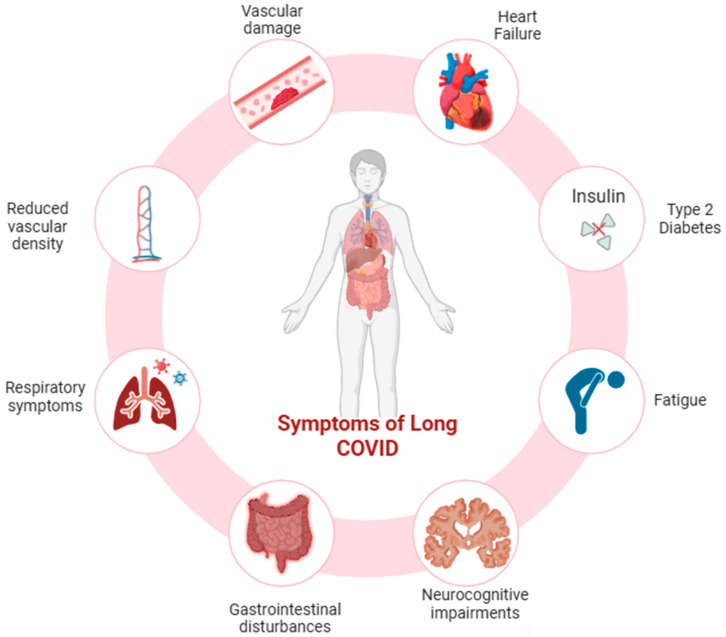
Systemic manifestations associated with long COVID. This illustration summarizes the major multi-organ symptoms reported in individuals with long COVID. Persistent respiratory symptoms (such as dyspnea, chronic cough, and reduced pulmonary function) coexist with reduced vascular density and vascular damage, reflecting endothelial dysfunction frequently documented after SARS-CoV-2 infection. Cardiac complications, including heart failure and arrhythmias, highlight the cardiovascular burden of long COVID. Metabolic disturbances, notably impaired insulin signaling and Type 2 diabetes, represent long-term consequences of post-viral metabolic dysregulation. Fatigue, one of the most prevalent symptoms, reflects systemic and mitochondrial involvement. Gastrointestinal disturbances (including abdominal pain, altered motility, and inflammation) and neurocognitive impairments (“brain fog,” memory issues, reduced concentration) underscore the multisystemic nature of the condition. Together, these interconnected manifestations illustrate the widespread and persistent physiological impact of long COVID. This figure is original and was created by the authors using BioRender and AI-assisted design tools.

The origins of long COVID are likely to involve various, potentially overlapping factors encompassing immune dysregulation [10], disruption of microbiota [11,12], autoimmunity [13], abnormal clotting and endothelial function [14], fibrosis, and dysfunctional neurological signaling [15]. As summarized in Figure 1, long COVID arises from the convergence of persistent immune dysregulation, endothelial injury, metabolic and mitochondrial dysfunction, fibrotic remodeling, and neuroinflammation, highlighting the multisystem nature of post-acute sequelae.

**Figure 2:**
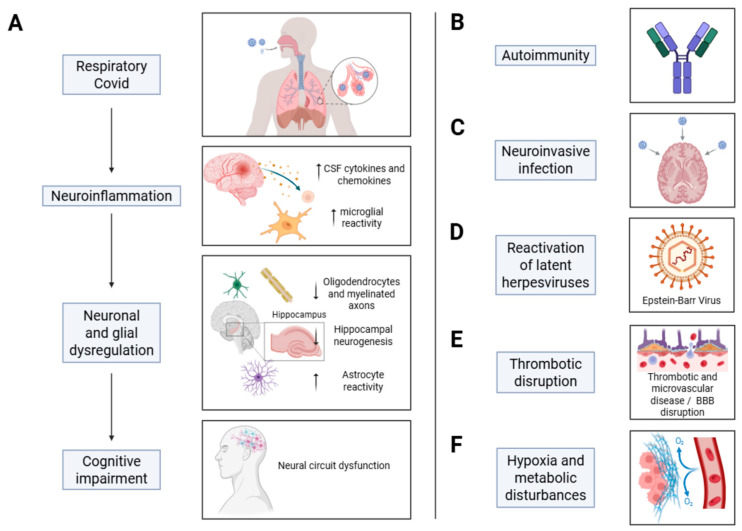
Possible mechanisms contributing to COVID-19-related cognitive impairment. (**A**) Respiratory SARS-CoV-2 infection induces systemic inflammation that can propagate to the CNS via circulating cytokines and chemokines, resulting in neuroinflammation. Elevated CNS inflammatory mediators activate microglia and astrocytes, disrupt myelin homeostasis and plasticity, impair hippocampal neurogenesis, and alter neural circuit function, thereby contributing to cognitive dysfunction. (**B**) Immune dysregulation may promote the generation of anti-neural autoantibodies and autoreactive T cells, leading to autoimmune encephalitis and sustained immune-mediated neural injury. (**C**) Direct neuroinvasion by SARS-CoV-2 is rare but has been reported in a limited number of cases. (**D**) SARS-CoV-2 infection can trigger reactivation of latent herpesviruses, particularly Epstein–Barr virus (EBV), which may further amplify neuroinflammatory responses. (**E**) Neurovascular pathology, including blood–brain barrier disruption, fibrinogen extravasation, and microvascular thrombosis, contributes to CNS inflammation and neuronal injury. (**F**) In severe COVID-19, hypoxia and metabolic disturbances secondary to pulmonary and multiorgan dysfunction can directly damage the nervous system and exacerbate cognitive impairment. This figure is original and was created by the authors using BioRender and AI-assisted design tools.

Key risk factors for long COVID consistently identified include sex, as females have significantly higher risk of developing long COVID [16], having pre-existing health conditions like cardiovascular disease, asthma, diabetes, and depressive disorders [17], as well as vaccination status, which is supported by recent meta-analyses from 2025, which found that unvaccinated individuals have over twice the odds of developing long COVID compared to vaccinated people [5], and the variant of SARS-CoV-2 with pre-Omicron variants carrying a higher risk [18]. These demographic, clinical, and virological risk factors are integrated in Figure 2, which illustrates how host susceptibility and infection-related variables shape long COVID prevalence and heterogeneity.

The clinical heterogeneity of long COVID is well recognized and poses significant challenges for diagnosis and treatment. Many patients encounter a multitude of symptoms affecting multiple organ systems [19]. Long COVID encompasses a range of adverse outcomes. Starting with vascular damage, endothelial dysfunction seen in long COVID patients contributes to thrombosis, pulmonary embolism, and bleeding [20,21]. Thrombosis is also caused by microclots found in both acute and long COVID [22]. Further, these patients show lasting changes in blood cell deformability and reduced vascular density, especially in small capillaries up to 18 months post-infection [23], and a persistent increase in cardiovascular risks (heart failure, arrhythmias, stroke) independent of the initial severity of the disease [24]. Long COVID is associated with new-onset type 2 diabetes [25], myalgic encephalomyelitis/chronic fatigue syndrome (ME/CFS), and dysautonomia, particularly postural orthostatic tachycardia syndrome (POTS). It is also associated with neurocognitive impairments, gastrointestinal disturbances, respiratory symptoms, fibrosis-related sequelae, and hormonal alterations [26].

This variability complicates clinical recognition and management, especially with the lack of standardized biomarkers or tests. This raises the need for validated biomarkers to improve personalized diagnosis and treatment approaches.

Many studies suggest that several microRNAs (miRNAs) are involved in many biological processes linked to the risk factors associated with long COVID. Thus, these small non-coding RNAs that regulate gene expression post-transcriptionally play crucial roles as regulators, biomarkers, and potential therapeutic targets in long COVID. In fact, recent studies have identified a set of dysregulated miRNAs in long COVID patients [27,28].

The prolonged dysregulation of miRNAs following SARS-CoV-2 infection may drive persistent inflammation, vascular dysfunction, altered cellular metabolism, and tissue remodeling processes, which are proposed to underlie the long-term manifestations of long COVID [29]. Because miRNAs regulate immunity, metabolism, and fibrosis, the three interconnected pillars of long COVID pathology, they represent key molecular players in this condition [30]. Through their influence on immune cell activation, cytokine signaling, mitochondrial function, and extracellular matrix turnover, miRNAs may orchestrate the transition to the chronic systemic dysfunction seen in long COVID and could serve as valuable biomarkers for long COVID.

Because miRNAs circulate in blood and other body fluids, they hold promise as non-invasive biomarkers for diagnosing and monitoring the progression of long COVID. Furthermore, targeting specific miRNAs represents a promising strategy for developing novel therapeutic approaches aimed at mitigating chronic symptoms in the affected patients.

It is noteworthy that this manuscript presents a narrative review intended to provide a conceptual and integrative overview of current evidence rather than a systematic or exhaustive synthesis. The relevant literature was identified through searches of PubMed, Scopus, and Web of Science, focusing on articles published between 2020 and 2025. Search terms included long COVID, post-acute sequelae of SARS-CoV-2 infection (PASC), microRNAs, immune dysregulation, inflammation, and related keywords. Additional studies were identified by manual screening of the reference lists of selected articles. Long COVID was defined according to the World Health Organization (WHO) definition. Article selection was based on relevance to the scope of the review and scientific quality.

While several recent reviews, including that of Paval et al. [29], have summarized dysregulated microRNAs and their potential diagnostic relevance in long COVID, the present review is distinguished by its integrative mechanistic focus. Rather than providing a descriptive catalogue, we systematically link validated miRNA–target interactions to persistent immune dysregulation, mitochondrial and metabolic dysfunction, endothelial injury, and fibrotic remodeling. In addition, this review uniquely emphasizes the therapeutic implications of miRNA dysregulation, including emerging miRNA-based interventions and delivery strategies, to highlight their potential as actionable targets in long COVID.

## 2. Overview of miRNA Biology

### 2.1. Definition

MicroRNAs (miRNAs) are endogenous short non-coding RNA molecules, typically 19 to 25 nucleotides long with a single-stranded structure. They play a key role in regulating gene expression post-transcriptionally [31]. They are highly conserved throughout evolution and are encoded in the genomes of nearly all eukaryotes [32]. Generally, miRNAs modulate the expression of their target genes via binding to complementary regions in the mRNA 3′ UTR, either promoting their degradation or inhibiting their translation. Studies have shown that individual genes can be subject to regulation by multiple miRNAs, and a single miRNA can also regulate multiple mRNA targets (up to 200) simultaneously [33]. As the field of miRNA research has developed, it has become clear that deviations in miRNA profiles play a critical role in various pathological processes, including neurological disorders, cardiovascular disease and cancer [34].

### 2.2. Biosynthesis

**Figure 3:**
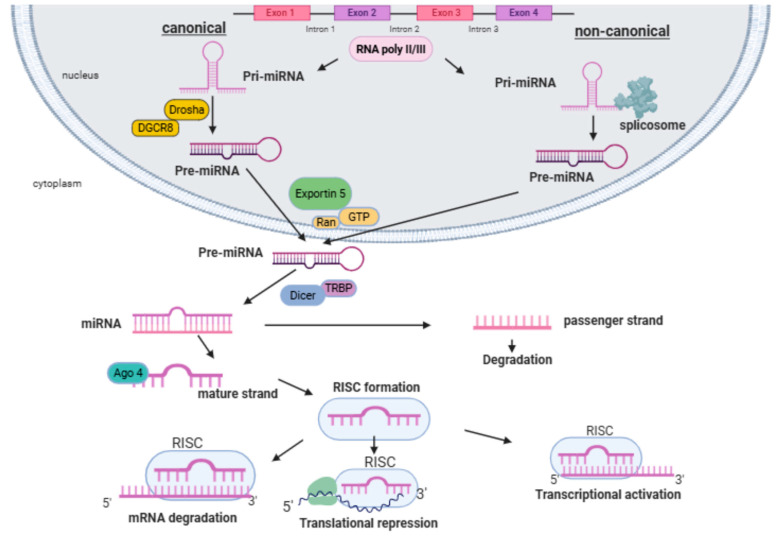
Exonic or intronic miRNAs undergo transcription by RNA polymerase II/III, resulting in the formation of a pri-miRNA. Within the nucleus, Drosha and DGCR8 collaborate to process the pri-miRNA into a pre-miRNA. Alternatively, in the non-canonical pathway, splicing mediated by a spliceosome replaces Drosha cleavage. The pre-miRNA is then transported to the cytoplasm with the assistance of exportin 5 and Ran/GTP. Once in the cytoplasm, Dicer and TRBP further refine the pre-miRNA. The passenger strand undergoes degradation, leaving the mature strand to associate with the Ago4 protein, forming the RISC complex. RISC primarily induces either mRNA degradation in the case of complete complementarity or translational repression in the case of incomplete complementarity. It is worth noting that the enzymes involved in this process include Drosha (RNase III endonuclease), DGCR8 (DiGeorge syndrome critical region 8), and Dicer (RNase III endonuclease).

MicroRNAs undergo a series of post-transcriptional processing steps as part of their biogenesis [35]. MiRNA genes are transcribed either from introns of protein-coding genes or by intergenic miRNAs under the control of their own promoters. Their synthesis begins in the nucleus, where hairpin-structured primary miRNAs (pri-miRNA) are transcribed by RNA polymerases II and III. These pri-miRNA (about 1000 bp in size) are cleaved by microprocessors, including the RNase III endonuclease DROSHA and RNA-binding protein cofactor DiGeorge syndrome critical region 8 (DGCR8), to produce 60 to 70 bp nucleotide stem loop precursor miRNAs (pre-miRNAs) [35]. The resulting pre-miRNAs are then transported to the cytoplasm by exportin 5, a transport protein on the nuclear membrane [36]. Once in the cytoplasm, the nuclease Dicer further processes the pre-miRNAs by cleaving them into short double-stranded miRNA duplexes. One strand of this duplex is degraded, and the other becomes a mature miRNA. This mature miRNA is loaded into the Argonaute (Ago2)-containing RNA-induced silencing complex (RISC), guiding gene silencing by targeting specific mRNA [36]. The canonical steps of miRNA biogenesis and post-transcriptional gene regulation are illustrated in Figure 3, providing a framework for understanding how miRNA dysregulation can exert broad systemic effects.

### 2.3. mRNA Silencing and Feedback Regulation

As mentioned earlier, once the miRNA binds to its target gene, it silences it through mRNA degradation or translation inhibition, where perfect complementarity results in mRNA cleavage and degradation while partial complementarity leads to translational repression or mRNA destabilization through deadenylation and decapping mechanisms [35].

Beyond silencing, miRNAs are also integral to feedback and feedforward regulatory loops [37,38]. For instance, transcription factors that activate a gene may simultaneously induce a miRNA that later suppresses that gene’s expression; this creates a negative feedback loop that fine-tunes protein output [39]. Conversely, feedforward loops occur when a transcription factor induces a miRNA, and both the transcription factor and the induced miRNA jointly influence the same downstream targets to reinforce a cellular state [40]. These dynamic feedback mechanisms are crucial for fine-tuning gene expression and ensuring homeostatic balance in cellular signaling and differentiation, as well as regulating biological processes. The disruption of this feedback control is a hallmark of different pathological conditions, including inflammation, fibrosis, cancer, and long COVID.

### 2.4. Circulating miRNAs: Stability in Serum/Extracellular Vesicles and Value as Biomarkers

Circulating miRNAs are remarkably stable in body fluids such as serum, plasma, saliva, cerebrospinal fluid, and urine. This is because they are protected within molecular carriers that shield the miRNA from degradation and facilitate their intercellular communication. These carriers can be extracellular vesicles (EVs) such as exosomes and microvesicles that encapsulate miRNAs in lipid bilayers, RNA-binding proteins like Argonaute 2 (Ago2) and nucleophosmin 1 (NPM1), or high-density lipoproteins (HDLs) that shuttle miRNAs between cells [41]. This structural protection allows for the detection and quantification of circulating miRNAs even under variable storage and handling conditions.

Most miRNAs exhibit strictly regulated expression patterns, usually tissue-specific or even cell-specific, highlighting the importance of miRNAs in the time, space, and developmental stages of specific gene expression patterns. So, understanding this pattern can help better understand the normal state and the disruption of the respective tissue [42]. This is why miRNAs are viewed as promising non-invasive biomarkers.

For example, distinct circulating miRNA signatures have been associated with cardiovascular diseases [43], neurodegeneration [44], cancer [45], and viral infections [46], including SARS-CoV-2 infection [47] and long COVID [29].

In this context, circulating miRNAs can provide insight into systemic pathophysiological processes that are proposed to underlie long COVID. Also, circulating miRNA profiles could help monitor disease progression, predict outcomes, and assess therapeutic responses, offering a bridge between molecular pathogenesis and clinical approaches.

## 3. Evidence of miRNA Dysregulation in Long COVID

### 3.1. Clinical Profiling Studies

Clinical profiling studies effectively demonstrated that convalescent patients retain a distinct plasma or serum miRNA pattern several months after infection. These persistent alterations represent a reproducible molecular signature in individuals with long COVID, reflecting ongoing states of unresolved inflammation, endothelial injury, and tissue remodeling.

In patient cohorts, the most frequently reported alterations effectively include downregulation of miR-146a and miR-29 family, alongside upregulation of miR-21 and miR-15 [29]. These verified clinical changes provide a molecular basis for the disease, and they regulate core inflammatory and fibrotic circuits. miR-146a restrains NF-κB signaling by targeting IRAK1 and TRAF6 [48]. In acute COVID-19, circulating miR-146a levels are significantly reduced, and this reduction correlates with elevated IL-6 concentrations and poor response to anti-IL-6 therapy [49,50]. Evidence in the post-acute/long COVID phase remains mixed: while some post-COVID cohorts show restored or even elevated miR-146a-5p expression during recovery [51], others, particularly cardiovascular comorbidity groups, report persistent downregulation associated with higher serum IL-6 [52]. miR-21 promotes cardiac fibrosis through the activation of the TGF-β1/Smad-3 signaling pathway. This pathway is crucial for the progression of fibrosis [53]. miR-155 is known for its role in regulating inflammatory responses and is often upregulated in inflammatory conditions, which can contribute to gut inflammation in long COVID [54], and the miR-29 family allows for the upregulation of these genes, facilitating the accumulation of fibrous connective tissue in the kidneys. TGF-β, a key mediator of extracellular matrix remodeling, during viral infections is elevated in plasma from patients with post COVID-19 sequelae [55]. Their persistent dysregulation thus provides a plausible molecular basis for chronic low-grade inflammation and progressive fibrosis in long COVID. A summary of these dysregulated miRNAs and their associated pathways is presented in Table 1.

Indeed, symptom clustering across cohorts has provided valuable insights into the relationship between molecular alterations and clinical phenotypes. In the fatigue-dominant cluster, the most common phenotype in long COVID, patients effectively show miRNA signatures of elevated miR-155 and miR-21 combined with reduced miR-146a, a molecular profile that perpetuates low-grade inflammation by sustained NF-κB and IL-6 pathway activation.

On the otherhand, research focusing on post-COVID patients exhibiting rheumatologic complaints has been found to have further persistent decreases in miR-200c-3p, miR-766-3p, and miR-142-3p, reinforcing the notion of sustained immune perturbation long after viral clearance [27]. Collectively, these findings indicate that the circulating miRNA landscape retains an “epigenetic memory” of the acute inflammatory episode, which may influence the trajectory of post-viral recovery.

In COVID-19 and convalescent cohorts, miRNAs such as miR-126-3p and miR-223-3p, identified in EV, remain effectively altered months after infection, paralleling endothelial and innate immune dysfunction [51]. Recent efforts have emphasized extracellular-vesicle (EV)-associated miRNAs as more reliable biomarkers than total plasma miRNAs. Encapsulation within vesicular membranes shields miRNAs from RNase degradation and pre-analytical variability, conferring greater stability and reproducibility [75]. Moreover, EVs selectively package miRNAs involved in immune, vascular, and metabolic signaling, allowing fine-grained discrimination between patient subgroups [76]. The enhanced robustness of EV-miRNAs suggests potential utility in longitudinal monitoring and therapeutic stratification in long COVID.

Overall, converging evidence from these clinical studies underscores that long COVID represents a sustained, miRNA-mediated imbalance in immune and fibrotic homeostasis. Integrating these circulating and EV-derived miRNA profiles with multi-omics and clinical phenotyping could yield powerful diagnostic and prognostic tools for patient management and targeted intervention.

### 3.2. Immune and Inflammatory Regulation

**Figure 4:**
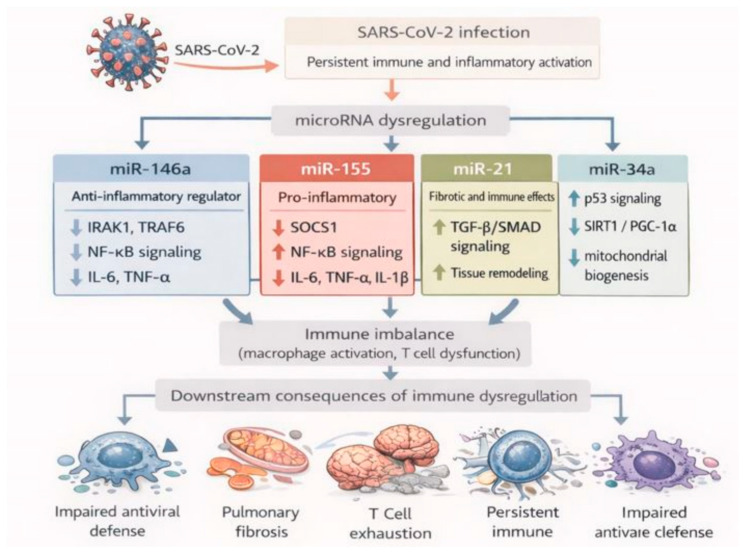
Immune and inflammatory regulation in long COVID. Persistent SARS-CoV-2-induced immune activation leads to dysregulation of key microRNAs involved in inflammatory control. miR-146a acts as a negative regulator of NF-κB signaling, while upregulation of miR-155 suppresses SOCS1 and amplifies pro-inflammatory cytokine production. Concurrently, miR-21 promotes fibrotic and immune-regulatory pathways via TGF-β/SMAD signaling. The combined effects of these alterations contribute to macrophage activation, T cell dysfunction, impaired antiviral defense, and downstream organ-specific complications observed in long COVID. This figure is original and was created by the authors using BioRender and AI-assisted design tools.

As depicted in Figure 4, dysregulated miRNAs modulate key inflammatory pathways, including NF-κB, cytokine signaling, and immune cell activation, thereby contributing to the persistence of chronic inflammation in long COVID. By acting as post-transcriptional modulators of gene expression, these miRNAs control signaling pathways involved in cytokine production, macrophage activation, interferon responses, and T-cell differentiation. Dysregulation of this miRNA network in convalescent patients appears to prolong inflammatory signaling long after acute SARS-CoV-2 infection, thereby contributing to the pathophysiological landscape of post-acute sequelae (long COVID).

Among the most consistently implicated miRNAs is miR-146a, a key negative regulator of innate immune signaling in plasma and serum samples from post-acute and long COVID patients. miR-146a levels have frequently been reported as reduced, particularly in individuals exhibiting persistent systemic inflammation and elevated IL-6 concentrations. Mechanistically, miR-146a directly targets interleukin-1 receptor-associated kinase 1 (IRAK1) and tumor necrosis factor receptor-associated factor 6 (TRAF6), thereby constraining Toll-like receptor and NF-κB signaling. Sustained downregulation of miR-146a removes this inhibitory brake, leading to prolonged NF-κB activation and continued production of pro-inflammatory cytokines such as IL-6 and TNF-α. This miRNA–target–pathway axis provides a mechanistic explanation for chronic inflammatory persistence observed in a subset of long COVID patients [77,78,79]. In contrast, miR-155 was found to be upregulated in acute COVID-19 and in the convalescent or post-acute phase of long COVID patients [80,81]. Mechanistically, miR-155 functions as a pro-inflammatory amplifier; it targets the suppressor of cytokine signaling 1 (SOCS1), a critical negative regulator of the JAK/STAT pathway. By repressing SOCS1, elevated miR-155 sustains cytokine-driven STAT activation, promoting macrophage activation, Th1/Th17 polarization, and continued NF-κB-dependent inflammatory transcription. Clinically, persistent miR-155 elevation has been associated with ongoing immune activation, endothelial dysfunction, and increased cardiovascular risk, linking immune dysregulation with systemic long COVID sequelae [81,82]. Further, miR-21 plays a multifaceted role at the intersection of immune regulation and tissue remodeling. In post-COVID and long COVID cohorts, miR-21 has been detected at elevated levels in circulating biofluids, particularly in patients with cardiopulmonary symptoms. Beyond its immunomodulatory effects on T-cell differentiation and STAT3 signaling, miR-21 directly targets SMAD7, thereby enhancing TGF-β/SMAD signaling. This mechanism establishes a molecular bridge between chronic inflammation and fibrotic remodeling, suggesting that persistent miR-21 dysregulation may contribute to the progression from immune activation to structural tissue damage in long COVID [83,84,85]. Other immune-modulatory miRNAs are also implicated in post-acute immune dysregulation. For example, the miR-29 family regulates interferon-stimulated genes and antiviral defence, and its downregulation in long COVID can impair viral clearance and immune homeostasis [86]. While not yet verified in all long COVID cohorts, members of the let-7 family, miR-31, miR-34a, miR-424/503, and miR-138 are strong mechanistic candidates for the persistent immune dysfunction observed in patients. These molecules have been shown in other viral contexts to modulate immune checkpoint pathways, T-cell exhaustion, and macrophage polarization, providing a theoretical framework for the immune dysfunction seen in long COVID, though direct patient measurements for some of these specific molecules are still emerging [87,88,89].

### 3.3. Mitochondrial and Metabolic Dysfunction

Mitochondrial dysfunction and altered metabolic homeostasis are increasingly recognized as important features associated with long COVID, potentially contributing to hallmark clinical manifestations, including chronic fatigue, post-exertional malaise, dysautonomia, and cognitive impairment. Accumulating evidence suggests that dysregulation of specific miRNAs may contribute mechanistically to these abnormalities through modulating mitochondrial biogenesis, oxidative phosphorylation (OXPHOS), reactive oxygen species (ROS) generation, and metabolic reprogramming.

Clinical observations of metabolic reprogramming in long COVID patients have been linked to the hypothesized persistence of miR-210. While miR-210 upregulation is well documented during acute SARS-CoV-2 infection, its persistence into the post-acute or chronic phase has been proposed as a potential contributor to the bioenergetic deficits and chronic fatigue frequently reported by patients [90,91]. miR-210 is often referred to as a hypoxamiR due to its induction under hypoxic and inflammatory conditions via HIF-1α and NF-κB signaling [92,93]. In post-acute and convalescent COVID cohorts, miR-210 has been reported as persistently upregulated in circulating plasma and extracellular-vesicle fractions, particularly in individuals with ongoing inflammatory and metabolic symptoms. Mechanistically, miR-210 directly targets ISCU (iron–sulfur cluster scaffold protein) and SDHD (succinate dehydrogenase complex subunit D), both essential for electron-transport-chain (ETC) function [92,93]. Sustained repression of these targets has been shown to impair oxidative phosphorylation and promote a shift toward glycolysis, resulting in reduced ATP production. In addition, miR-210 upregulation has been associated with increased mitochondrial ROS generation and a pseudo-hypoxic state even under normoxic conditions, processes that are consistent with mechanisms implicated in chronic fatigue and autonomic dysfunction observed in long COVID [91,94]. However, direct causal links in post-acute patient tissues remain to be established.

Another important regulator is miR-34a, a stress-responsive microRNA transcriptionally induced by p53 and pro-inflammatory cytokines such as IL-6 and TNF-α [95]. Elevated miR-34a levels have been detected in circulating biofluids and endothelial extracellular vesicles during and after SARS-CoV-2 infection, with EV-associated miR-34a linked to post-COVID clinical outcomes, including metabolic dysregulation [27]. miR-34a directly targets SIRT1, a NAD^+^-dependent deacetylase that activates PGC-1α, a central regulator of mitochondrial biogenesis and antioxidant defense [96]. By repressing SIRT1, miR-34a diminishes PGC-1α activity, leading to reduced mitochondrial biogenesis, accumulation of dysfunctional mitochondria, and impaired mitophagy [97]. Persistent miR-34a dysregulation suggests a potential link between persistent inflammatory stress and long-term mitochondrial insufficiency in long COVID.

A subset of miRNAs, known as mitomiRs, localize directly to mitochondria where they regulate mitochondrial gene expression and electron-transport-chain (ETC) activity. For instance, miR-181c translocates into mitochondria and targets COX1, a key component of complex IV, thereby modulating ETC efficiency and reactive oxygen species (ROS) generation [98]. Other mitomiRs, including miR-181c, miR-499, and miR-378, are discussed as mechanistic factors that may contribute to the fragmented mitochondria and oxidative stress potentially underlying long COVID. miR-181c regulates mitochondrial function by fine-tuning mitochondrial membrane potential, ROS production, and ATP synthesis, thereby modulating the activation of inflammatory signaling pathways [98]. miR-499 and miR-378 have been shown to regulate mitochondrial dynamics, for example, miR-499 targets the Drp1-dependent fission machinery and thereby maintains the mitochondrial network [99], and miR-378a-3p has been implicated in controlling fusion/fission balance and mitophagy [100]. Therefore, dysregulation of these mitomiRs has been proposed as a contributing factor to mitochondrial fragmentation, oxidative stress, and metabolic exhaustion observed in post-viral syndromes, including long COVID [101].

Another important aspect is that mitochondrial dysfunction and chronic inflammation reinforce one another in the setting of long COVID. Persistent cytokine signaling, including IL-6 and TNF-α, can induce miRNAs such as miR-210 and miR-34a, perpetuating mitochondrial impairment [91]. In turn, mitochondrial reactive oxygen species (ROS) production triggers activation of the NF-κB pathway and inflammasome pathways (e.g., NLRP3), which sustain cytokine release and establish a self-amplifying inflammatory metabolic feedback loop. This framework may help explain why metabolic and inflammatory abnormalities persist in some individuals long after viral clearance [102,103].

Clinically, these molecular alterations provide a mechanistic basis for many long COVID symptoms. Bioenergetic failure reduces exercise tolerance and contributes to post-exertional malaise; oxidative stress in neurons is proposed to underlie cognitive deficits and dysautonomia. Moreover, impaired mitochondrial metabolism in immune cells may sustain chronic activation and hinder immune resolution [91].

Therapeutically, targeting miRNAs involved in mitochondrial dysfunction for example, inhibiting miR-210, which represses the iron–sulfur cluster assembly proteins ISCU1/2 and disrupts electron-transport-chain function [104], or miR-34a, which targets SIRT1 and diminishes PGC-1α-dependent mitochondrial biogenesis, has been suggested to play a role, and restoration of SIRT1/PGC-1α activity has been proposed as a potential strategy to improve mitochondrial performance and alleviate symptoms.

**Figure 5:**
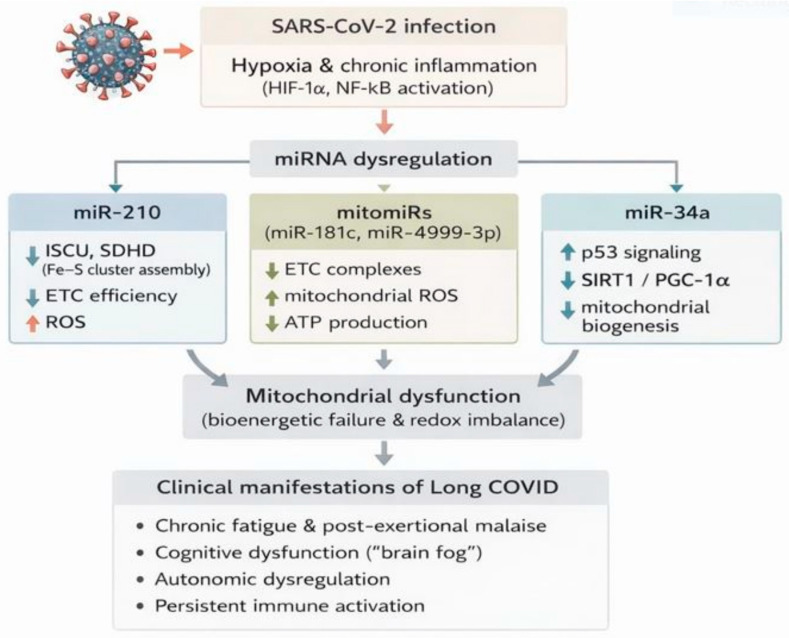
Mitochondrial dysfunction and bioenergetic failure in long COVID driven by SARS-CoV-2-induced miRNA dysregulation and chronic inflammation. Persistent infection triggers hypoxia-inducible Factor-1α (HIF-1α) and NF-κB inflammatory pathways, leading to miRNA dysregulation: miR-210 upregulates PDH (pyruvate dehydrogenase) inhibition and impairs SDH (succinate dehydrogenase) in the electron transport chain (ETC) assembly, while increasing reactive oxygen species (ROS); mitochondrial miR-499-5p downregulates ETC complexes and ATP production alongside ROS elevation; miR-34a suppresses SIRT1/PGC-1α signaling critical for mitochondrial biogenesis. These converge on core mitochondrial failure and redox imbalance, manifesting clinically as cognitive fatigue (“brain fog”), post-exertional malaise, and autonomic dysregulation. This figure is original and was created by the authors using BioRender and AI-assisted design tools.

Modulation of mitomiRs such as miR-181c, which controls mitochondrial-encoded COX1 and affects membrane potential and ROS balance [98], may also restore electron-transport-chain integrity and reduce oxidative stress, highlighting miRNAs as both biomarkers and potential therapeutic targets in long COVID [91]. These miRNA-mediated effects on mitochondrial bioenergetics and metabolic reprogramming are summarized in Figure 5, linking altered miRNA expression to fatigue, post-exertional malaise, and dysautonomia.

Although direct longitudinal mitochondrial measurements in long COVID tissues remain limited, the convergence of circulating miRNA profiling, validated miRNA–target interactions, and consistent symptom associations supports a mechanistic role for miRNA-mediated mitochondrial dysfunction. These findings position mitochondrial-regulatory miRNAs as both biomarker candidates and therapeutic targets, offering a mechanistic framework linking immune activation to metabolic failure in long COVID [90,91]. Where direct post-acute patient data are lacking, the mechanistic interpretations presented in this section are derived in part from acute COVID-19 cohorts or related disease models and should therefore be regarded as hypothesis-generating rather than definitive.

### 3.4. Endothelial Injury and Fibrosis

**Figure 6:**
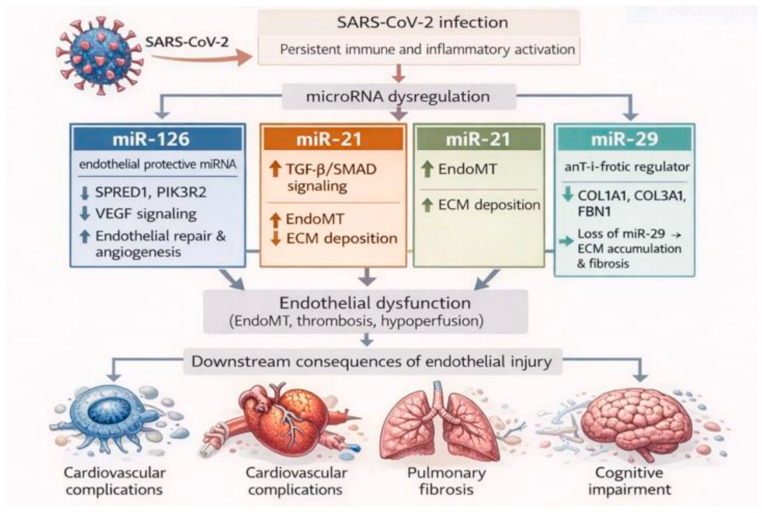
Endothelial injury and fibrotic remodeling in long COVID. Persistent SARS-CoV-2-induced endothelial injury and inflammation drive dysregulation of key microRNAs involved in vascular homeostasis and fibrosis. Reduced miR-126 impairs VEGF signaling and endothelial repair, promoting microvascular dysfunction and thrombosis. Concurrent upregulation of miR-21 activates TGF-β/SMAD signaling, inducing endothelial-to-mesenchymal transition (EndoMT) and extracellular matrix (ECM) deposition. Loss of miR-29 removes inhibitory control over collagen and fibrillin expression, resulting in unchecked fibrotic remodeling. These combined mechanisms contribute to persistent vascular dysfunction, pulmonary fibrosis, cardiovascular complications, and cognitive impairment in long COVID. This figure is original and was created by the authors using BioRender and AI-assisted design tools.

Endothelial dysfunction has emerged as a central pathological feature of long COVID, contributing to persistent cardiovascular, pulmonary, and neurological complications, including microvascular thrombosis, impaired tissue perfusion, and chronic inflammation. miRNAs play a central role in regulating vascular homeostasis, angiogenesis, extracellular matrix (ECM) turnover and fibrotic signaling. Dysregulation of these small non-coding RNAs has been increasingly implicated in the persistence of endothelial injury and pathological tissue remodeling characteristic of long COVID-related vascular disease [29]. Figure 6 integrates miRNA-driven pathways involved in endothelial dysfunction, angiogenic impairment, and fibrotic remodeling, illustrating mechanisms underlying vascular and tissue injury in long COVID.

Among the endothelial-specific miRNAs, miR-126 has been most strongly associated with vascular integrity and repair. miR-126 promotes angiogenesis and endothelial regeneration by targeting negative regulators of the VEGF signaling pathway, including SPRED1 and PIK3R2 [56,105]. In patient cohorts, a characteristic decrease in miR-126-3p has been effectively identified, paralleling persistent endothelial dysfunction. Evidence from acute COVID-19 and convalescent studies suggests this downregulation persists in long COVID patients, serving as a marker for ongoing vascular injury and pro-thrombotic risk [29]. Persistent miR-126 suppression may impair endothelial cell proliferation and migration, delay vascular repair, and promote a pro-thrombotic and pro-inflammatory endothelial phenotype. These alterations are consistent with microvascular rarefaction, chronic tissue hypoperfusion, and increased long-term cardiovascular risk phenomena increasingly recognized in long COVID cohorts [29,106].

In parallel, in convalescent COVID and long COVID cohorts, elevated circulating miR-21 has been associated with cardiopulmonary symptoms. In endothelial cells, miR-21 promotes endothelial-to-mesenchymal transition (EndoMT) by enhancing TGF-β/SMAD signaling, in part through repression of SMAD7. EndoMT leads to loss of endothelial identity, reduced nitric oxide bioavailability, and increased fibrotic signaling, thereby linking endothelial dysfunction to progressive vascular and tissue remodeling [107].

Conversely, the miR-29 family, well known for its anti-fibrotic properties, is consistently down-regulated in severe COVID-19 and post-acute disease states. miR-29 directly represses multiple ECM-related genes, including COL1A1, COL3A1 and FBN1, thereby acting as a brake on collagen synthesis and tissue fibrosis [108,109]. Its suppression removes this regulatory constraint, allowing uncontrolled ECM accumulation and fibrotic scarring. Importantly, the opposing regulation of miR-21and miR-29 has been effectively documented in post-acute disease states. Patients effectively exhibit an upregulation of miR-21 alongside a downregulation of miR-29, creating a verified pro-fibrotic molecular environment that mirrors the miRNA signature in idiopathic pulmonary fibrosis (IPF) [29,109]. This overlap suggests shared fibrogenic mechanisms between IPF and post-COVID-19 fibrosis and supports the conceptual relevance of targeting these miRNAs therapeutically.

The clinical consequences of endothelial injury and fibrosis in long COVID are wide-ranging. Persistent endothelial dysfunction has been associated with microvascular thrombosis, chronic inflammation, and elevated cardiovascular risk, including accelerated atherosclerosis and myocardial damage [110]. In the lungs, fibrotic remodeling leads to interstitial scarring, decreased compliance, and impaired gas exchange, often manifesting as chronic dyspnoea and exercise intolerance. Similarly, microvascular damage in the brain has been implicated in cognitive impairment and “brain fog.” Collectively, these pathologies significantly reduce quality of life and contribute to the multisystem nature of long COVID.

Therapeutic modulation of miRNAs represents a promising yet largely preclinical avenue for addressing these sequelae. Restoration of miR-126 expression could enhance endothelial repair and angiogenesis [56], while antagonising miR-21 might suppress fibrotic signaling. Moreover, miR-29 mimics such as MRG-229 have suggested efficacy in reducing fibrosis in preclinical lung models [109], highlighting the potential of miRNA-based interventions in long COVID. As research advances, miRNA signatures could serve not only as diagnostic and prognostic biomarkers but also as targets for precision therapies aimed at mitigating endothelial damage and fibrotic remodeling in post-COVID patients.

Where direct long COVID endothelial tissue data are limited, mechanistic interpretations derived from acute COVID-19 and related vascular disease models are explicitly framed as hypothesis-generating. The consistency of circulating and extracellular-vesicle—associated miRNA alterations, together with validated miRNA—target interactions, supports a potential contributory role of miRNA dysregulation in the persistence of endothelial dysfunction in long COVID, rather than definitive causality

## 4. Circulating miRNAs as Biomarkers: Diagnostic and Prognostic Potential

The search for reliable biomarkers capable of distinguishing long COVID from full recovery or predicting long-term outcomes has intensified in recent years. Among the various molecular candidates, circulating miRNAs, both free and extracellular-vesicle (EV)-associated, have emerged as particularly promising tools. Their stability in plasma and serum, resistance to RNase degradation, and close regulation of immune and endothelial signaling make them attractive for post-acute biomarker development. Although current studies remain small and heterogeneous, an increasingly coherent picture is forming in which altered miRNA expression reflects the lingering immune and vascular perturbations that define the post-COVID condition [27,51,111] (Table 2).

Early investigations using integrative multi-omics approaches have demonstrated that the circulating miRNA landscape remains perturbed months after viral clearance. In a cohort of convalescent individuals, approximately two months after recovery, clinically, studies have identified a characteristic decrease in miR-126-3p and miR-223-3p, coupled with a relative increase in miR-146a-5p, in exhaled breath condensates compared with healthy controls [51]. These alterations paralleled persistent endothelial and inflammatory dysfunction and correlated with clinical indices of reduced physical performance during rehabilitation. The observed downregulation of miR-126-3p, a key endothelial homeostasis regulator [112], and miR-223-3p, a modulator of innate immune activation, supports the notion of ongoing microvascular and immune dysregulation as molecular hallmarks of long COVID [58].

Consistent with these findings, plasma miRNAs in post-COVID patients presented rheumatologic manifestations several months after infection. They reported sustained decreases in miR-200c-3p, miR-142-3p, and miR-766-3p, alongside altered IgG-mediated hydrolysis of selected miRNAs, indicating that immune-mediated mechanisms continue to shape the post-viral transcriptomic milieu [27]. These patterns reinforce the hypothesis that miRNAs act as epigenetic “memory traces” of the acute inflammatory phase, potentially driving chronic immune dysregulation in susceptible individuals.

Extracellular-vesicle studies further strengthen the biomarker rationale. EVs protect miRNAs from degradation and selectively package cargo relevant to immune and vascular signaling. Elevated endothelial EV-associated miR-34a in convalescent patients predicted the development of new-onset diabetes, highlighting the prognostic utility of vesicular miRNAs for post-COVID complications [111].

Collectively, these emerging datasets suggest that distinct miRNA patterns may distinguish long COVID from full recovery and provide mechanistic insights into symptom persistence. Decreased miR-126-3p and miR-223-3p point to endothelial and innate immune activation, while elevated miR-146a-5p likely reflects compensatory anti-inflammatory feedback within the NF-κB axis [27,51,111]. Although these findings hold diagnostic promise, current evidence is limited by small cohort sizes (often <50 participants), variable definitions of long COVID, and a lack of standardized analytical pipelines. No single miRNA or panel has yet achieved validated diagnostic or prognostic cut-offs with high sensitivity and specificity.

Future studies must therefore focus on multi-center, phenotype-resolved cohorts with standardized sampling times and normalization methods to translate these signals into clinically actionable biomarkers. Integrating circulating and EV-associated miRNA profiles with cytokine, proteomic, and metabolomic data could enable the creation of multidimensional signatures predictive of recovery trajectories or specific complications. In sum, while the field remains at an early stage, circulating miRNAs, particularly miR-126-3p, miR-223-3p, and miR-146a-5p, represent promising diagnostic and prognostic candidates that capture the enduring molecular imprint of SARS-CoV-2 infection in long COVID [27,51,111].

Despite their promise, several translational barriers currently limit the clinical implementation of circulating miRNAs as biomarkers for long COVID. Reported miRNA signatures exhibit substantial inter-study variability, reflecting cohort heterogeneity, differences in disease definition and severity, and variability in biospecimen types and analytical platforms. In addition, the lack of standardized normalization strategies and validated diagnostic cut-offs complicates cross-study comparison and clinical interpretation. Finally, considerable overlap between miRNA profiles reported in long COVID and those observed in other chronic inflammatory and post-viral conditions poses challenges for disease specificity, underscoring the need for large, well-phenotyped longitudinal cohorts and harmonized analytical frameworks.

## 5. Therapeutic Targeting of miRNAs in Long COVID

### 5.1. AntagomiRs to Dampen Persistent Inflammation

It is important to note that the miRNA-based therapeutic strategies discussed in this section are primarily supported by preclinical studies, acute COVID-19 models, or related inflammatory and fibrotic diseases. Their relevance to long COVID remains inferential and should be interpreted as conceptual, hypothesis-generating frameworks intended to guide future experimental and clinical investigation rather than established therapeutic evidence.

AntagomiRs are synthetic analogs of miRNAs that function as silencing agents that inhibit specific miRNAs. They are used in the development of new therapeutics for the regulation of different gene expression in disease states [113] (Table 3).

Preclinically, several antagomiRs have been used to reduce pathogenic inflammation and fibrosis, including those involved in long COVID. miR-155 is a key regulator of immune cell development and function, and its upregulation contributes to uncontrolled inflammation and organ injury as seen in different infections such as sepsis [114]. Its inhibition in animal models has been shown to reduce inflammatory cytokine levels and improve survival outcomes, thus showing therapeutic potential for controlling excessive inflammation in systemic infections. In long COVID, upregulation of miR-155 may contribute to inflammation and immune dysregulation through its upregulation. In SARS-CoV-2-infected hACE2 transgenic mice, the treatment with anti-miR-155 was shown to improve survival and reduce lung damage and inflammatory cytokine levels [115].

miR-21 is a critical regulator promoting lung fibrosis through its actions on pulmonary fibroblasts. In a study by Gang Liu et al., it was shown that during lung fibrotic diseases such as idiopathic pulmonary fibrosis (IPF), miR-21 is upregulated primarily in myofibroblasts, where it enhances fibrogenic activity. The use of anti-miR-21 attenuated these effects, thus suggesting therapeutic potential in fibrotic lung diseases [116]. Similar effects are seen with the upregulation of miR-21 in long COVID through the modulation of different key inflammatory and immune signaling pathways [29]. The use of antagomiRs targeting miR-21 may help alleviate long COVID symptoms by reducing fibrosis and inflammation, similar to their proven effectiveness in other fibrotic lung diseases.

Moreover, miR-34a, which is known to contribute to excessive inflammatory responses by regulating key pro-inflammatory cytokines such as TNF-α and IL-6, and suppressing anti-inflammatory cytokines, like IL-10, has been proven to be upregulated in endothelial extracellular vesicles during COVID-19 infection. This elevated level is linked to lung injury and metabolic dysregulations, particularly new-onset diabetes, which is one of the important metabolic sequelae seen in long COVID patients [111]. The miR-34a antagomir’s effect observed in sepsis-induced lung injury models, especially via reducing inflammation [117], can be conceptually extrapolated to its potential role in long COVID.

Similarly, miR-181a expression elevation has been shown to promote inflammation and neuronal damage in COVID-19 severe cases, potentially contributing to long COVID pathology [132]. miR-181 antagomirs’ proven ability to attenuate this induced inflammation by reducing NF-κB activation and infiltration of immune cells into the brain, cell death, and improve neurological deficits in stroke models [118] suggests potential as therapeutic agents to mitigate neurological and inflammatory sequelae in long COVID.

These data support the idea that antagomiRs could be repurposed to blunt sustained inflammatory and fibrotic pathways that contribute to long COVID sequelae. Among these, miR-155 and miR-21 have been directly validated in SARS-CoV-2 models, whereas miR-34a and miR-181a remain candidate targets extrapolated from related inflammatory and fibrotic conditions, requiring further validation in long COVID cohorts.

### 5.2. miRNA Mimics Restore Immune Homeostasis

miRNA mimics are synthetic, double-stranded RNA molecules designed to imitate endogenous miRNAs in cells, thus increasing the gene-silencing activity of specific miRNAs by binding to target mRNAs and causing their repression, either by inhibiting translation or promoting degradation [119]. In this way, miRNA mimics restore or augment the function of endogenous miRNAs that are downregulated during disease. In cases of inflammation, many anti-inflammatory miRNAs that favor excessive innate immune signaling (e.g., TLR/IRAK/TRAF6 → NF-κB), promote anti-inflammatory macrophage polarization, reduce cytokine storms, protect barrier function, and favor resolution of inflammation [133] can be replaced with mimics to relieve persistent inflammation, such as in the case of persistent immune dysfunction after SARS-CoV-2 infection. In fact, several miRNAs have been suggested to exert pro-inflammatory effects in preclinical models, making them candidates for exploration in long COVID.

For instance, miR-146a relieves inflammation as it attenuates NF-κB activation and downstream cytokine production via targeting IRAK1 and TRAF6. In miR-146a-deficient mice, which normally exhibit hyperactive NF-κB signaling and inflammation, the intravenous injections of myeloid-targeted miR-146a mimic reduced cytokine storms in models of cytokine release syndrome and bacterial hypersensitivity and modulated inflammation [121]. Despite being upregulated in long COVID, the endogenous miR-146a response may be insufficient or dysregulated to fully control the persistent inflammation seen; therefore, the use of miR-146a mimics can enhance or restore this regulatory function to get the desired anti-inflammatory effect similar to the effects seen in other models with persistent inflammation [29,122].

Other anti-inflammatory miRNAs are also plausible candidates for mimic-based therapy to restore immune homeostasis in Long COVID. Human post-COVID profiling studies have reported a decrease in miR-223 and miR-126 expression, both of which act as key negative regulators of inflammation by targeting cytokine production and immune cell activation pathways. Preclinical and translational data show that miR-223 mimics attenuate pulmonary and systemic inflammation in sepsis models [120], and miR-126 mimics reduce inflammation and oxidative stress in endothelial cells subjected to injury [134]. The anti-inflammatory effects demonstrated by miR-126 and miR-223 mimics in multiple experimental inflammation models provide promising therapeutic potential for long COVID, which shares similar underlying inflammatory processes.

Moreover, miR-124, which plays a critical role in mediating the cholinergic anti-inflammatory pathway by targeting signal transducer and activator of transcription 3 (STAT3) to reduce IL-6 production and by targeting TNF-α converting enzyme (TACE) to decrease TNF-α release, has demonstrated protective effects in multiple animal models of sepsis and acute lung injury when delivered as a mimic [123]. MiR-124 expression has been reported to be decreased in patients with long COVID, particularly in relation to neuroinflammatory processes [68].

Other candidates with anti-inflammatory roles include miR-150, which primarily suppresses the production of pro-inflammatory cytokines such as IL-1β, IL-6, and TNF-α in macrophages. The use of miR-150 mimics has been shown to contribute to reduced inflammation in LPS-stimulated THP-1 macrophage cells [124]. Its expression in COVID-19 is reported to be reduced, contributing to sustained inflammation and impaired immune resolution in the long term, so the use of mimics to compensate for its diminished anti-inflammatory role could be considered.

Similarly, miR-181b, which is reported to suppress NF-κB-mediated vascular inflammation in preclinical studies, is also dysregulated in COVID-19 [125], and there is potential for the use of its mimics as therapeutic agents to prevent the persistent inflammation seen in long COVID.

In summary, several anti-inflammatory miRNAs have preclinical evidence that their mimics can blunt pathogenic inflammation. These could serve as promising candidates to alleviate the persistent inflammation observed in COVID-19 cases and their long-term sequelae. Importantly, all of the miRNAs highlighted in this section have been validated as dysregulated in long COVID patient cohorts, supporting their consideration as therapeutic targets rather than speculative candidates.

## 6. Delivery Strategies: Lipid Nanoparticles (LNPs) and Exosome Engineering

Efficient delivery of miRNAs remains a major challenge when it comes to multi-system conditions such as long COVID. Two of the most promising routes are lipid nanoparticles (LNPs) and engineered exosomes or extracellular vesicles (EVs), both of which have demonstrated significant progress in both preclinical and early translational studies [126,127].

Lipid nanoparticles (LNPs) have now been adapted for miRNA mimics and antagomiRs delivery. These are clinically validated non-viral vectors for delivering nucleic acids such as mRNA vaccines [127]. Lipid nanocarriers protect miRNAs from enzymatic degradation and enable targeted delivery by improving cellular uptake through electrostatic interactions between their positively charged lipid components and the negatively charged miRNAs. Their modular design allows fine-tuning of lipid composition, surface charge, and PEGylation, which can be optimized to improve tissue-specific targeting, enhance endosomal escape, and increase circulation stability [126]. This versatility makes lipid nanocarriers a highly adaptable platform for efficient and safe miRNA delivery in diverse therapeutic contexts.

Lipid nanoparticle (LNP) delivery of miR-126, miR-223, and miR-181b mimics has shown significant anti-inflammatory effects in preclinical models of vascular inflammation, sepsis, and lung injury [56,135,136,137].

AnatgomiRs have also been effectively delivered by nanoparticles in inflammatory and ischemic disease models, demonstrating reduced tissue damage and inflammation, such as those of miR-125b and miR-155 [130,131]. While direct studies on LNP-miRNA delivery in long COVID remain limited, these examples underscore the therapeutic potential of using LNPs to deliver anti-inflammatory miRNA mimics to regulate immune dysregulation and persistent inflammation characteristic of long COVID.

Exosome or extracellular-vesicle (EV) engineering offers another route for miRNA delivery. Exosomes are endogenous lipid vesicles secreted by most cells that mediate intercellular communication by transferring proteins, lipids, and RNAs (including miRNAs). Being bound to the lipid bilayer membrane, exosomes can provide protection to miRNAs against enzymatic degradation, allowing stable circulation in the body and cell-specific targeting via receptor-mediated endocytosis or direct membrane fusion. They possess low immunogenicity and high biocompatibility since they are derived from host cells. These properties make them inherently promising as vehicles for delivering therapeutic miRNAs, whether mimics or antagmoiRs, to target tissues [138].

Exosomes are engineered for miRNA delivery through different approaches. One method involves genetically modifying the parent cells to produce exosomes that are naturally enriched with specific miRNAs or surface targeting molecules, thus facilitating controlled cargo loading during biogenesis. Alternatively, isolated exosomes can be directly loaded with desired miRNAs using physical techniques such as electroporation, sonication, or freeze–thaw cycles, which transiently permeabilize the exosome membrane, allowing therapeutic miRNAs to be encapsulated. Chemical modifications to exosome surfaces by conjugating targeting ligands such as RVG, integrins, and peptides can enhance miRNA loading efficiency and targeting precision. These engineering strategies allow for efficient therapeutic miRNA transfer [139].

Exosomes have been used for delivering miRNAs that target inflammation and other underlying processes relevant to long COVID. For instance, exosomal miR-146a and miR-155 are reported to regulate inflammation by modulating immune cell responses and cytokine production [140].

Moreover, bone marrow mesenchymal stem cell-derived extracellular vesicles (EVs) miR-23b delivery to the injured spinal cord reduces inflammation and promotes tissue repair by lowering cytokine production, limiting microglial activation, and enhancing recovery [66]. Another study showed that the delivery of this same miRNA—miR-23b—via mannose-modified exosomes alleviates acute lung injury by inhibiting pro-inflammatory macrophage activation through the NF-κB pathway [67]. Other miRNAs like miR-145-5p carried in exosomes have demonstrated anti-inflammatory effects by regulating TLR4/NF-κB signaling and inflammasome activity in neuroinflammatory models [141].

Among the miRNA targets discussed, those with existing clinical or late preclinical development pipelines appear most realistic for near- to mid-term translation. In particular, anti-miR-21- and miR-29-based strategies, already advanced in fibrotic and cardiovascular disease models [57], as well as miR-126 modulation for endothelial repair, represent the most mature candidates based on current evidence [56]. In contrast, other miRNA targets remain at an earlier exploratory stage and primarily serve to define mechanistic pathways and future therapeutic directions.

Despite encouraging preclinical progress, miRNA-based therapeutic approaches for long COVID remain speculative, and their clinical translation will likely require long-term validation in well-designed longitudinal and interventional studies

## 7. Future Perspective

Despite rapid progress, further research into the role of miRNAs in long COVID remains essential. Future studies must move beyond descriptive profiling toward establishing causal relationships between specific miRNA alterations and defined long COVID symptoms and trajectories. Large, multi-center longitudinal cohorts with standardized sampling, analytical pipelines, and harmonized clinical phenotyping will be critical to track miRNA dynamics over time and to distinguish recovery from progression to chronic disease. Such approaches will enable the identification of robust, reproducible miRNA signatures with prognostic value.

Integration of miRNA profiling with other omics layers, such as proteomics, metabolomics, and immune phenotyping, represents an important next step. Multi-omics approaches will allow construction of integrated regulatory networks linking miRNAs to downstream molecular pathways, immune dysfunction, metabolic failure, and tissue injury, thereby strengthening mechanistic inference and biomarker reliability.

Moreover, it is essential to determine the cellular and tissue origins of circulating miRNAs, including contributions from immune cells, endothelial cells, skeletal muscle, and the nervous system. Single-cell and spatial transcriptomic approaches, combined with extracellular-vesicle characterization, will be instrumental in clarifying organ-specific sources of dysregulated miRNAs. This will improve understanding of how systemic and organ-restricted processes interact to drive the heterogeneous clinical manifestations of long COVID.

Deeper exploration of the interactions between host miRNAs and viral RNAs may reveal previously unrecognized mechanisms of viral persistence, immune evasion, or sustained immune dysregulation. Characterizing miRNA–viral RNA interactions across different tissues and disease stages could uncover novel therapeutic entry points aimed at restoring immune balance and limiting chronic inflammation or residual viral activity.

Finally, the translation of miRNA research into clinical practice will require rigorous validation of miRNA biomarkers across independent cohorts, as well as careful evaluation of miRNA-based therapeutic strategies, including mimics, antagomiRs, and targeted delivery systems such as lipid nanoparticles and engineered extracellular vesicles. If successfully validated, miRNA-based diagnostics and therapeutics have the potential to transform long COVID care by enabling earlier diagnosis, improved disease monitoring, and personalized treatment strategies tailored to individual molecular profiles.

## 8. Conclusions

Long COVID is a complex, multisystem disorder driven by persistent immune, endothelial, metabolic, and fibrotic dysregulation following SARS-CoV-2 infection. This review highlights accumulating evidence that miRNAs represent key post-transcriptional regulators underlying these long-term abnormalities, with durable alterations in circulating and extracellular-vesicle-associated miRNA profiles observed months after recovery. These miRNA signatures provide mechanistic insight into disease persistence and hold promise as non-invasive biomarkers for patient stratification and prognosis. Although clinical translation remains in its early stages, miRNA-based therapeutic strategies, including mimics and antagomiRs, offer a rational framework for mechanism-guided interventions. Well-designed longitudinal studies will be essential to validate miRNA biomarkers and advance their integration into precision approaches for Long COVID management.

## Figures and Tables

**Table 1:** Key dysregulated circulating microRNAs in long COVID and their associated pathways.

miRNA	Expression Variation in Long COVID	Validated Molecular Target(s)	Sample	Key Pathways Affected	Evidence Type	Associated Long COVID Phenotype(s)	References
miR-146a	Downregulated	IRAK1, TRAF6	Plasma, blood, exhaled breath condensate	Anti-inflammatory: TLR signaling, NF-κB pathway, IL-6 regulation (reported in sepsis/inflammation models)	Post-acute COVID cohorts; extrapolated mechanistic models	Persistent inflammation, fatigue	[48,51]
miR-223	Downregulated	NLRP3	Plasma, exhaled breath condensate	Anti-inflammatory: NLRP3 inflammasome, neutrophil activation, IL-1β production	Acute and post-acute COVID	Intestinal immune balance and epithelial integrity, gastrointestinal dysfunction	[56,57]
miR-21	Mixed, often downregulated	PTEN, SMAD7	Plasma, blood, exosomes	Pro-fibrotic and pro-inflammatory: NF-κB, STAT3, fibrosis, IFN modulation	Acute COVID; extrapolated fibrotic models	Cardiovascular complication, pulmonary fibrosis	[58,59,60]
miR-155	Upregulated in acute COVID; dysregulated long COVID	SOCS1	Plasma, serum, PBMC	Pro-inflammatory: SOCS1, NF-κB, JAK2/STAT3 pathways	Acute and post-acute COVID	Pulmonary and gastrointestinal complications	[61]
miR-29 (family)	Dysregulated; often reduced in long COVID fibrosis reports	TGF-β1/Smad, (PI3K/Akt/mTOR	Plasma, PBMC	Anti-fibrotic: TGF-β signaling, ECM remodeling	Acute and post-acute COVID	Renal, pulmonary and gastrointestinal dysfunction	[62,63]
miR-126	Downregulated	SPRED1	Exhaled breath condensate, plasma, endothelial exosomes	Anti-inflammatory and anti-fibrotic: endothelial function, angiogenesis, inflammation	Post-acute COVID cohorts	Endothelial dysfunction and contribute to vascular complications	[63]
miR-181b	Downregulated	Importin-α3, MAPK	Plasma, blood	Anti-inflammatory: NF-κB signaling, vascular inflammation	Acute and post-acute COVID	Vascular inflammation	[64]
miR-150	Downregulated	AKT2, CXCR4	Plasma, blood	Anti-inflammatory: immune response regulation, inflammation	Post-COVID follow-up cohorts	lung injury, immune dysregulation	[65]
miR-23b	Downregulated	NF-κb	Plasma, preclinical EV, lung tissue	Anti-inflammatory: NF-κB pathway, macrophage activation	Immune/endothelial models (extrapolated to long COVID)	Persistent inflammation, endothelial dysfunction	[66,67]
miR-124	Downregulated	STAT3, CEBP-α	Blood, neural tissue models	Anti-inflammatory: neuroinflammation, immune signaling	Post-acute COVID	Gastrointestinal symptoms such as motility disorders and altered gut–brain communication, neurodegenerative diseases, and cognitive dysfunction	[68,69,70]
miR-200c-3p	Downregulated	ZEB1, ZEB2	Plasma	Pro-fibrotic and pro-inflammatory: EMT activation by targeting ZEB1, ZEB2	Post-COVID clinical cohorts	Inflammation, immune dysregulation, and impaired tissue repair	[27,71]
miR-142-3p	Downregulated	TGF-β	Plasma	Anti-inflammatory; regulates immune responses and TGF-β signaling, IL-1/IL-6 signaling	Post-COVID	Persistent inflammation, immune dysregulation	[27,29,72]
miR-766-3p	Downregulated	STAT3, TGF-β	Plasma	Anti-inflammatory and anti-fibrotic: STAT3, TGF-β pathway, NF-κB signaling	Post-COVID cohorts	Persistent inflammation, fibrosis	[27,73,74]

**Table 2:** Circulating miRNAs as candidate biomarkers in long COVID.

Cohort Size and Population	Long COVID Definition/Phenotype	Follow-Up Time	Biospecimen	Analytical Platform	Key miRNA Findings	Clinical/Mechanistic Associations	Major Limitations	Reference
Small cohort (<50); convalescent adults vs. healthy controls	Persistent post-COVID symptoms with reduced physical performance during rehabilitation	~2 months after acute recovery	Exhaled breath condensate	qRT-PCR–based miRNA profiling	↓ miR-126-3p, ↓ miR-223-3p, ↑ miR-146a-5p	Endothelial dysfunction, persistent inflammation	Small sample size; non-blood biospecimen; limited symptom stratification; cross-sectional design	[51]
Small cohort (<50); patients with long COVID rheumatological symptoms vs. COVID-19 recovered patients without complaints	Persistent rheumatological symptoms post-acute SARS-CoV-2 infection.	Several months post-infection	Plasma	qRT-PCR and immunochemical assays	↓ miR-200c-3p, ↓ miR-142-3p, ↓ miR-766-3p; Altered IgG-mediated miRNA hydrolysis	Ongoing inflammation and endothelial dysfunction, contributing to rheumatological symptoms	Small sample sizes No causation proven between miRNA/antibody changes and symptoms	[27]
Small convalescent cohort metabolic follow-up	Post-COVID patients without diabetes at baseline	Months after recovery	Endothelial extracellular vesicles	EV isolation + miRNA profiling (qRT-PCR)	↑ EV-associated miR-34a	Predicted new-onset diabetes; links endothelial dysfunction to metabolic sequelae	Limited cohort size; single complication focus; needs external validation	[111]

**Table 3:** microRNA-based therapeutic strategies in long COVID.

Therapeutic Strategy	miRNA	Main Molecular Targets/Pathways	Mechanism of Action	Relevance to Long COVID Pathology	References
AntagomiRs (miRNA inhibition)	miR-155	SOCS1, SHIP1, NF-κB signaling	Inhibition of miR-155 reduces pro-inflammatory cytokine production (TNF-α, IL-6), dampens immune overactivation, and limits tissue injury	Persistent immune activation, cytokine elevation, lung inflammation	[114,115]
	miR-21	PTEN, SMAD7, TGF-β signaling	Anti-miR-21 attenuates fibroblast activation and fibrotic remodeling	Pulmonary fibrosis, chronic inflammation	[29,116]
	miR-34a	IL-6, TNF-α, metabolic signaling pathways	AntagomiR reduces excessive inflammation and endothelial dysfunction	Lung injury, metabolic sequelae (e.g., diabetes)	[117]
	miR-181a	NF-κB, neuronal inflammatory pathways	AntagomiR decreases neuroinflammation, immune cell infiltration, and neuronal damage	Neurocognitive impairment, neuroinflammation	[118]
miRNA mimics (functional restoration)	miR-146a	IRAK1, TRAF6 → NF-κB	Mimics restore negative feedback on innate immune signaling and suppress cytokine storms	Persistent inflammation, immune dysregulation	[119]
	miR-223	NLRP3 inflammasome, IL-1β	Mimics suppress macrophage activation and systemic inflammation	Pulmonary and systemic inflammation	[120]
	miR-126	VCAM-1, oxidative stress pathways	Mimics protect endothelial function and reduce vascular inflammation	Endothelial dysfunction, microvascular injury	[119,121,122]
	miR-124	STAT3, TACE (TNF-α processing)	Mimics activate cholinergic anti-inflammatory pathway and reduce IL-6 and TNF-α	Neuroinflammation, systemic inflammation	[123]
	miR-150	IL-1β, IL-6, TNF-α	Mimics suppress macrophage-driven inflammation	Sustained immune activation	[124]
	miR-181b	NF-κB-mediated vascular inflammation	Mimics reduce endothelial and vascular inflammation	Vascular dysfunction	[125]
Lipid nanoparticles (LNPs)	miR-126, miR-223, miR-181b (mimics); miR-155, miR-125b (antagomiRs)	NF-κB, cytokine signaling, endothelial pathways	Protect miRNAs from degradation, enhance cellular uptake, enable targeted delivery	Multisystem inflammation, immune dysregulation	[126,127,128,129]
Engineered exosomes/EVs	miR-146a, miR-155, miR-23b, miR-145-5p	NF-κB, TLR4, inflammasome pathways	Natural vesicle-mediated delivery with low immunogenicity and tissue targeting	Persistent inflammation, lung and neuroinflammatory injury	[130,131]

## Data Availability

No new data were created or analyzed in this study.

## Abbreviations

Abbreviations: IRAK1: interleukin-1 receptor-associated kinase 1; TRAF6, tumor necrosis factor receptor-associated factor 6; NLRP3, NOD-, LRR-, and pyrin domain-containing protein 3; PTEN, phosphatase and tensin homolog; SMAD7, SMAD family member 7; SOCS1, suppressor of cytokine signaling 1; TGF-β1, transforming growth factor-β1; PI3K, phosphoinositide 3-kinase; Akt, protein kinase B; mTOR, mechanistic target of rapamycin; SPRED1, sprouty-related EVH1 domain-containing protein 1; Importin-α3, importin subunit alpha-3; MAPK, mitogen-activated protein kinase; AKT2, AKT serine/threonine kinase 2; CXCR4, C-X-C motif chemokine receptor 4; NF-κB, nuclear factor kappa B; STAT3, signal transducer and activator of transcription 3; CEBP-α, CCAAT/enhancer-binding protein alpha; ZEB1, zinc finger E-box-binding homeobox 1; ZEB2, zinc finger E-box-binding homeobox 2. Evidence Categories: acute COVID, data derived from patients during the acute phase of SARS-CoV-2 infection; post-acute COVID, data derived from patients weeks to months after infection, consistent with long COVID/PASC; post-COVID cohorts, human clinical cohorts assessed after recovery from acute infection; post-COVID follow-up cohorts, longitudinal human studies monitoring patients over time after COVID-19; post-COVID clinical cohorts, clinically characterized patient cohorts evaluated in the post-acute phase; extrapolated mechanistic models, evidence inferred from in vitro, animal, immune, endothelial, or non-COVID disease models and considered hypothesis-generating rather than long COVID-specific. Abbreviations: SOCS1: suppressor of cytokine signaling 1; SHIP1, Src homology 2-containing inositol phosphatase 1; NF-κB, nuclear factor kappa B; PTEN, phosphatase and tensin homolog; SMAD7, SMAD family member 7; TGF-β, transforming growth factor-β; IL-6, interleukin-6; TNF-α, tumor necrosis factor alpha; IRAK1, interleukin-1 receptor-associated kinase 1; TRAF6, tumor necrosis factor receptor-associated factor 6; NLRP3, NOD-, LRR-, and pyrin-domain-containing protein 3; IL-1β, interleukin-1 beta; VCAM-1, vascular cell adhesion molecule-1; STAT3, signal transducer and activator of transcription 3; TACE, TNF-α-converting enzyme; TLR4, Toll-like receptor 4.
